# Deep learning for end-to-end kidney cancer diagnosis on multi-phase abdominal computed tomography

**DOI:** 10.1038/s41698-021-00195-y

**Published:** 2021-06-18

**Authors:** Kwang-Hyun Uhm, Seung-Won Jung, Moon Hyung Choi, Hong-Kyu Shin, Jae-Ik Yoo, Se Won Oh, Jee Young Kim, Hyun Gi Kim, Young Joon Lee, Seo Yeon Youn, Sung-Hoo Hong, Sung-Jea Ko

**Affiliations:** 1 Department of Electrical Engineering, Korea University, Seoul, South Korea; 2 Department of Radiology, The Catholic University of Korea, Seoul, South Korea; 3 Department of Urology, The Catholic University of Korea, Seoul, South Korea

**Keywords:** Computed tomography, Renal cell carcinoma, Cancer imaging

## Abstract

In 2020, it is estimated that 73,750 kidney cancer cases were diagnosed, and 14,830 people died from cancer in the United States. Preoperative multi-phase abdominal computed tomography (CT) is often used for detecting lesions and classifying histologic subtypes of renal tumor to avoid unnecessary biopsy or surgery. However, there exists inter-observer variability due to subtle differences in the imaging features of tumor subtypes, which makes decisions on treatment challenging. While deep learning has been recently applied to the automated diagnosis of renal tumor, classification of a wide range of subtype classes has not been sufficiently studied yet. In this paper, we propose an end-to-end deep learning model for the differential diagnosis of five major histologic subtypes of renal tumors including both benign and malignant tumors on multi-phase CT. Our model is a unified framework to simultaneously identify lesions and classify subtypes for the diagnosis without manual intervention. We trained and tested the model using CT data from 308 patients who underwent nephrectomy for renal tumors. The model achieved an area under the curve (AUC) of 0.889, and outperformed radiologists for most subtypes. We further validated the model on an independent dataset of 184 patients from The Cancer Imaging Archive (TCIA). The AUC for this dataset was 0.855, and the model performed comparably to the radiologists. These results indicate that our model can achieve similar or better diagnostic performance than radiologists in differentiating a wide range of renal tumors on multi-phase CT.

Kidney cancer is one of the 10 most common cancers, and by far the most common type of kidney cancer is renal cell carcinoma (RCC), which occurs in 9 out of 10 cases of all kidney cancer^1^. According to 2016 World Health Organization statistics, the three major subtypes of RCCs are clear cell RCC (ccRCC), papillary RCC (pRCC), and chromophobe RCC (chRCC), which account for 90% of all RCCs, while the majority of benign renal tumors are angiomyolipoma (AML) and oncocytoma^2^. In the retrospective study of 916 patients who underwent partial nephrectomy for presumed RCC from preoperative imaging, 129 (14.1%) patients revealed benign pathology on the final diagnosis, including 66 (51.2%) oncocytomas and 37 (28.7%) AMLs^3^. To avoid unnecessary biopsy or surgery, it is important to accurately differentiate benign tumors from malignant ones in preoperative images^4–7^. Moreover, since treatment planning and prognosis prediction are highly dependent on the pathological subtype of renal tumor, it is required to correctly classify tumor subtypes in images.^8–11^. Multi-phase abdominal computed tomography (CT) is often used for detection and evaluation of renal tumors^8,9,12^. Typically, multi-phase CT is analyzed on the basis of the enhancement characteristics of the tumors^13^. However, there are strong overlaps in image-level features between renal tumor subtypes, which make subtype classification difficult and cause inter-observer variation^9^. These clinical challenges point to the need to develop automatic systems that can reduce misdiagnosis and inter-observer variation^14^.

Recently, deep learning based on convolutional neural networks (CNNs) has shown promising results on several medical image analysis tasks^15–18^. For renal lesions, deep learning has been applied to tumor segmentation^19–21^ and classification^4,22–24^. However, in most prior studies on tumor classification, lesions were classified into only two classes (benign/malignant)^4,22,23^ or the three RCC classes (ccRCC, pRCC, and chRCC)^24^. Moreover, the previous diagnosis systems required the manual lesion identification process, in which the regions of tumors are drawn by radiologists.

To overcome these limitations, we designed and evaluated an end-to-end deep learning framework for the classification of renal tumor subtypes into five classes including both benign and malignant tumors using multi-phase abdominal CT scans as the input data (Fig. 1). We investigated the performance of six radiologists in differential diagnosis of renal tumors and compared our deep learning model with the radiologists. We integrated tumor segmentation and subtype classification into a unified framework for the diagnosis solely on CT data without manual intervention, improving its practical utility.

Our framework first extracts the kidney and tumor masks from the whole CT volume for each phase using the three-dimensional (3D) CNN-based segmentation model. We obtained voxel-level segmentation labels to train this model. Then, the CT volumes of different phases are aligned based on the segmented regions, and finally, the CNN-based classification model analyzes the aligned tumor regions and predicts the subtype. Postoperative pathology-confirmed tumor labels were used to train the classification model.

In this study, we constructed a large dataset consisting of 1035 CT images from 308 patients who underwent nephrectomy for renal tumors between 2003 and 2020. This dataset contains five major subtypes of renal tumors including both benign and malignant tumors: oncocytoma, AML, chRCC, pRCC, and ccRCC, where all tumors in the dataset have been pathologically confirmed by surgery. We randomly selected 50 cases with at least three CT phases to test the model, and the rest of the cases were used for the training. Patient demographics, the distribution of kidney tumor subtypes, tumor size, and CT phases for training/testing are summarized in Table 1. For each patient, multiple phases were acquired at different times such as non-contrast, arterial (20–30 s after contrast injection), portal (60–70 s), and delayed (>180 s) phases. We collected voxel-level segmentation labels for each CT scan, where trained annotators manually delineated kidneys and tumors in the images and then a radiologist (experience of 11 years) refined the annotations. Supplementary Table 1 shows the manufacturers and model names of the CT scanners used in the training and test sets.

**Table 1:** Patient demographics, subtype, and tumor size distributions for training/test dataset.

	Total	Training set	Test set
Patients (*n*)	308	258	50
Age (years)			
−40	29	23	6
40–50	73	62	11
50–60	96	80	16
60–70	71	57	14
70–	39	36	3
Gender			
Female	167	140	27
Male	141	118	23
Subtype			
ccRCC	66	54	12
pRCC	69	58	11
chRCC	68	58	10
AML	60	51	9
Oncocytoma	45	37	8
Tumor size (cm)			
1–2	79	66	13
2–3	84	71	13
3–4	56	51	5
4–5	33	27	6
5–6	29	21	8
6–	27	22	5
CT phases			
Four phase	183	145	38
Three phase	66	54	12
Two phase^a^	46	46	–
Single phase^a^	13	13	–

^a^The single-phase and two-phase CT scans are used only for training our segmentation model.

On the test dataset, we compared the diagnostic performance of the model to six board-certified radiologists (average experience of 14 years, ranging 5–24 years). The radiologists independently reviewed the multi-phase CT scans of the test cases and had access to the patient’s age and gender, while this information was not provided to the model. The radiologists were instructed to provide up to two differential diagnoses. When the radiologist was sufficiently confident with the first diagnosis, the second diagnosis was not provided. Performance of radiologists was measured using the first diagnosis (top-1 performance) and using both the first and second diagnoses (top-2 performance).

**Fig. 1:**
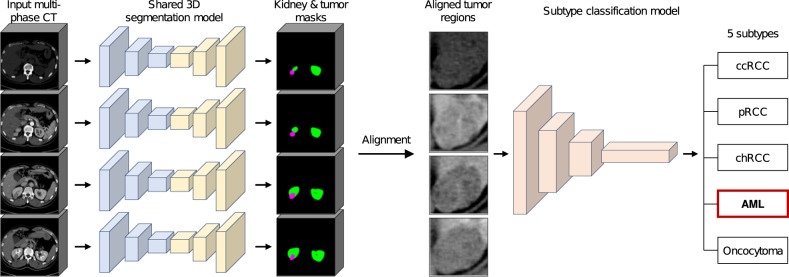
Overall deep learning framework. Our framework takes a multi-phase CT scan as an input. The framework first produces the kidney and tumor masks for each phase using a shared 3D segmentation model. The framework then aligns the tumor regions across phases and outputs a probability distribution over five subtype classes of renal tumor through a classification model. In the segmentation results, the green and magenta represent the kidney and tumor, respectively.

Figure 2a shows the receiver operating characteristic (ROC) curves of the model and the performance of the radiologists. We calculated the area under the curves (AUCs) with 95% confidence interval (CI) for each curve. The model achieved an average AUC of 0.889 (95% CI, 0.827–0.945), and exceeded both the top-1 and top-2 performance of the radiologists in most cases. In particular, the points indicating the average performance of the radiologists fell on or below the ROC curves of the model for all subtype classes. See Supplementary Fig. 1 for the precision–recall curves of the model. Figure 2b shows the confusion matrices for the model and all individual radiologists. We observed that chRCC, AML, and oncocytoma were frequently misclassified as ccRCC by the radiologists, whereas they were more correctly classified by the model. The model achieved the accuracy of 0.72, exceeding both the average top-1 and top-2 accuracy of radiologists, which were 0.42 and 0.56, respectively. Compared to the average radiologist, the model demonstrated statistically significant improvements in top-1 sensitivity (*P* < 0.05) for chRCC and AML, and even in top-2 sensitivity (*P* < 0.05) for AML (Fig. 2c). Also, there were statistically significant improvements in specificity (*P* < 0.05) for ccRCC and oncocytoma (Fig. 2d).

**Fig. 2:**
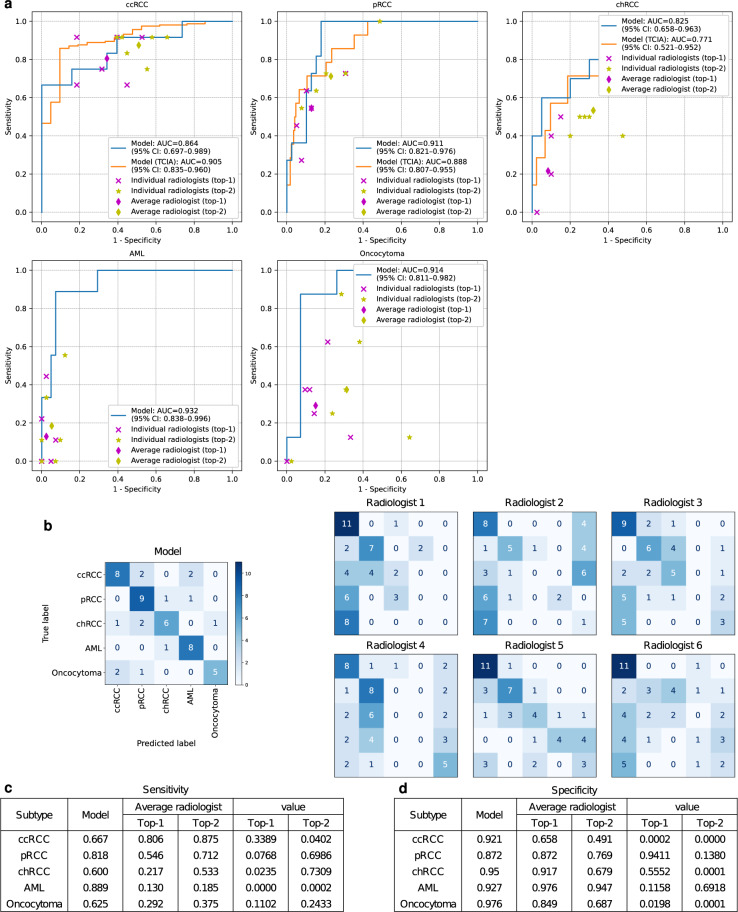
Kidney cancer diagnosis performance of the model and radiologists. **a** The ROC curves of the model and the performance of the six radiologists are plotted for each tumor subtype. For the three RCCs, the results of testing the model on the TCIA dataset are also plotted. **b** Confusion matrices for the model and individual radiologists. Comparison of the sensitivities (**c**) and specificities (**d**). *P* values were calculated using the two-sided permutation test.

To explore the generalizability of our model to different populations, we evaluated the model on an independent test dataset from The Cancer Imaging Archive (TCIA)^25^, which is a large public repository for research on cancer images. We collected 184 multi-phase CT scans of patients with renal tumors (163 ccRCC, 14 pRCC, and 7 chRCC). The cases of oncocytoma and AML are not available in this repository. We included cases with at least three CT phases for the study. The ROC curves on this test set are shown in Fig. 2a. The model achieved the average AUC of 0.855 (95% CI, 0.763–0.940), and the accuracy of 0.64. See Supplementary Figs. 2 and 3 for the precision–recall curves and the confusion matrix of the model. These results demonstrated that the model trained on the data collected from our hospital generalizes to the independent test set from different populations.

For the diagnostic performance comparison with radiologists, 40 cases (19 ccRCC, 14 pRCC, and 7 chRCC) were reviewed by the six radiologists. Supplementary Tables 2 and 3 provide the patient demographics, the number of individual tumor subtypes and CT phases, and the manufacturers and model names of the CT scanners for the full and radiologist-reviewed test sets. The ROC curves of the model and the performance of the radiologists are presented in Fig. 3a. The model achieved an average AUC of 0.863 (95% CI, 0.753–0.954) and performed on par with the radiologists. The points for the top-1 and top-2 performance of the average radiologist fell below the ROC curves of the model for pRCC and chRCC classes. Figure 3b shows the confusion matrices of the model and all individual radiologists. We observed that at least five chRCC cases were misclassified by radiologists, while only three chRCC cases were missed by the model. The model achieved the accuracy of 0.75, which exceeded the average top-1 accuracy of radiologists (0.63) and was slightly lower than the top-2 accuracy of radiologists (0.79). The model showed statistically significant improvement in top-1 sensitivity (*P* = 0.0112) for chRCC class compared to the average radiologist (Fig. 3c).

**Fig. 3:**
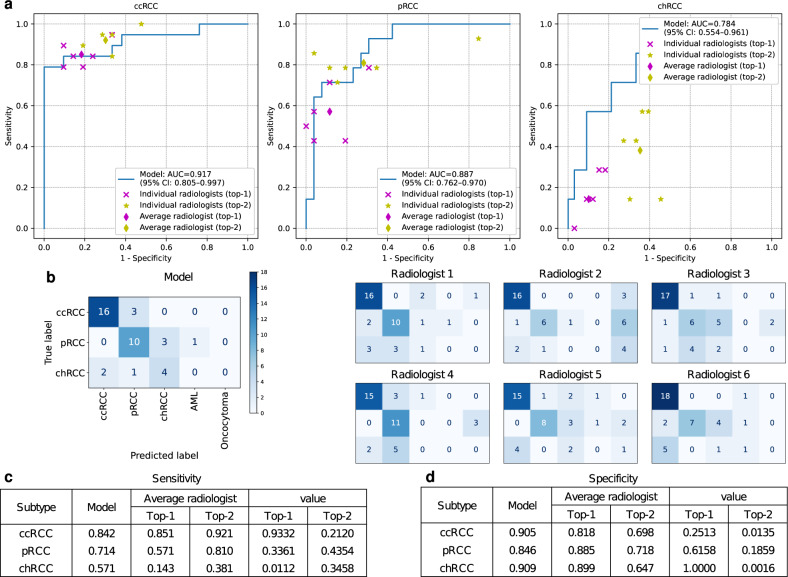
Performance comparison on an independent test dataset. **a** The ROC curves of the model and the performance of radiologists on the dataset from The Cancer Imaging Archive (TCIA) are plotted. This dataset contains three RCC subtypes. **b** Confusion matrices for the model and individual radiologists. Comparison of the sensitivities (**c**) and specificities (**d**). *P* values were calculated using the two-sided permutation test.

We also evaluated the performance of our segmentation and multi-phase registration models on the test dataset. First, we evaluated the segmentation model by measuring the Dice similarity coefficient (DSC)^26^, which quantifies the volume overlap between manual annotations and the masks produced by the model for the kidney and tumor regions. The average DSCs for the kidney and tumor were obtained as 0.969 ± 0.014 and 0.856 ± 0.131, respectively, while the DSCs for individual phases are presented in Supplementary Table 4a. The DSC for the tumor was higher than 0.87 in all phases except for the non-contrast phase. Second, we evaluated the registration model by measuring the DSC between the manual segmentation labels from the reference phase (portal phase) and the aligned labels from the other phases. The model achieved average DSCs of 0.934 ± 0.028 and 0.854 ± 0.092 for the kidney and tumor, respectively, which were much higher than those obtained by simply aligning the center of mass of the kidney volume (0.909 ± 0.053 and 0.770 ± 0.160). The results for all phases are summarized in Supplementary Table 4b.

There are several limitations of our study. First, the patients included in our dataset were only from Seoul St. Mary’s Hospital. Although we verified the performance from the external TCIA dataset as well as the separated internal test set, data collection from multiple centers in different countries is needed to train and test our model on more diverse populations. Second, we investigated the classification performance for the five renal tumor subtypes in this study. It would be beneficial to classify a wider range of subtype classes for the diagnosis, e.g., differentiating between type 1 pRCC and type 2 pRCC.

This study demonstrates that an end-to-end deep learning model can achieve radiologist-level performance for kidney cancer diagnosis using CT data. The proposed model successfully performed fine-grained classification of renal tumor into five major pathological subtypes including benign and malignant tumors. These results highlight the potential for fully automated systems to assist radiologists in diagnosing kidney cancer patients. Further studies with larger numbers of cases will be needed to validate the applicability of the model in clinical practice. In addition, we believe the presented deep learning framework could also be extended for the analysis of other cancer types and other modalities such as magnetic resonance imaging and positron emission tomography.

## Methods

### Dataset

Patients who underwent nephrectomy for renal tumor between 2003 and 2020 in Seoul St. Mary’s hospital were eligible. Among them, we selected 308 patients who underwent abdominal CT scans at Seoul St. Mary’s Hospital or other hospitals within 3 months before surgery. The CT scans were obtained with various imaging protocols and scanners. A radiologist reviewed all images and confirmed that the image quality was acceptable. Subtype labels were confirmed by pathological examination of the surgically removed tumors. All participants provided informed consent. This study was approved by the Seoul St. Mary’s Hospital Institutional Review Board. The slice thickness used was 5 mm in the majority of cases (71.2%) but could vary from 1 to 7 mm, and pixel spacing used ranged from 0.53 to 0.94 mm. To obtain pixel-level segmentation labels, 10 annotators supervised by a radiologist (experience of 11 years) first delineated the kidneys and tumors in the CT images, and these annotations were all checked and refined by the radiologist.

We also used image data from TCIA for validation of the model on an independent dataset. TCIA is a large public archive of cancer images where image data are contributed by multiple clinical institutions. We collected multi-phase CT scans of patients with RCC from The Cancer Genome Atlas kidney renal clear cell carcinoma (TCGA-KIRC)^25,27^, kidney renal papillary cell carcinoma (TCGA-KIRP)^25,28^, and kidney chromophobe (TCGA-KICH)^25,29^ databases. The results shown here are in whole or part based upon the data generated by the TCGA Research Network: http://cancergenome.nih.gov/. Cases for oncocytoma and AML were not available in TCIA. Only patients with three or more CT phases were included. The final dataset used consists of 600 CT images from 184 patients with the majority of tumor subtypes being ccRCC (163 cases). The TCIA data were only used for model testing. The slice thickness of the CT scans was 3 or 5 mm in most cases (535 scans), while the pixel spacing ranged from 0.54 to 0.98 mm. Supplementary Tables 2 and 3 describe the patient demographics, the number of individual subtypes and CT phases, and scanner information of this dataset.

### Model development

The proposed model has three main components: kidney and tumor segmentation, multi-phase alignment, and tumor subtype classification. All network components were implemented using the PyTorch framework^30^. The models were trained on an NVIDIA Titan Xp graphics processing unit (GPU). Data processing and analysis were performed using the Python language with the NiBabel, numpy and sklearn packages. ITK-Snap^31^ software was used for manual segmentation in CT volumes.

Recently, many deep learning-based semantic segmentation methods have been developed, such as FCN^32^, U-Net^33^, Deeplab V3+^34^, and PSPNet^35^. According to the kidney tumor segmentation challenge (KiTS19) reports^36^, the 3D U-Net architecture^37^ achieved the top performance over other methods. Hence, we adopted the 3D U-Net for kidney and tumor segmentation, where the network classifies each voxel in a CT volume into three classes: background, kidney, and tumor. This network was trained on 848 CT scans including four different contrast phases. The CT volumes were resampled to a 1.5 × 1.5 × 3 mm^3^ voxel size. The network parameters were then optimized using stochastic gradient descent on the sum of the cross-entropy and Dice loss function^38^. The hyperparameters required for training, such as the batch size and learning rate, were chosen by following nnU-Net^21^. This component produces the segmentation masks of the kidney and tumor for each phase of the CT volume.

We utilized 3D spatial transformer networks^39^ to register the multi-phase CT volumes. The 3D affine transformation parameters were optimized for each pair of volumes. We selected the portal phase as the reference phase, and registered the volumes from the other phases to the reference phase. If the portal phase was not available, the arterial phase was used instead for reference. The transformation parameters were iteratively updated to align the kidney and tumor masks of the two phases until convergence. We minimized the Dice loss using an Adam optimizer^40^ with a learning rate of 0.01. This registration component outputs the precisely aligned CT volumes of the non-reference phases.

We used ResNet-101 (ref. ^41^) to classify the pathological subtypes of renal tumor. For each case, the slice with the largest segmented tumor area was extracted from each phase of the CT scans, and the rectangular region containing the tumor region was then cropped from each extracted slice. The cropped images were then resized to 224 × 224 pixels and concatenated to form a 3-channel image, which was used as the input to the classification network. Cases with less than three CT phases were not used for training. For cases with four-phase CT scans, three 3-channel images were obtained by excluding each one of the three contrast-enhanced phases (arterial, portal, and delayed). These 3-channel images were used independently for the training. In the testing stage, we averaged the results of the network from three 3-channel images. We initialized ResNet-101 with the weights pre-trained on ImageNet^42^, and added a 1 × 1 × 1 convolutional layer at the beginning of the network and changed the last fully connected layer to produce a distribution over five classes. We trained the network using the cross-entropy loss with stochastic gradient descent. The final component outputs the probability for each subtype class.

### Stastical analysis

We computed confidence intervals for the AUC using 1000 bootstrap samples. We used a permutation test to compare the performance (sensitivity and specificity) of the model with that of the radiologists. The model predictions were randomly swapped with the radiologist decisions for each case, and the performance difference between the model and radiologist was calculated. This procedure was repeated 10,000 times, and then an empirical two-sided *P* value was obtained by comparing the observed performance difference with the empirical distribution of the differences.

### Reporting summary

Further information on research design is available in the Nature Research Reporting Summary linked to this article.

## Supplementary information

Supplementary Information

Reporting Summary

## Data Availability

The TCIA dataset used for the external validation is publicly available at the TCIA data portal (https://www.cancerimagingarchive.net). The dataset from Seoul St. Mary’s Hospital was used under approval for the current study. Restrictions apply to the availability of this dataset and so it is not publicly available. However, data are available from the authors upon reasonable request and with permission of Seoul St. Mary’s Hospital.

## Code availability

Our source code is available at https://github.com/khuhm/deep-kidney-cancer.
