# Supplementary material for: Deep learning for end-to-end kidney cancer diagnosis on multi-phase abdominal computed tomography
Source: NPJ Precis Oncol. 2021 Jun 18;5:54. doi: 10.1038/s41698-021-00195-y (PMC8213852; doi:10.1038/s41698-021-00195-y)
Supplement: Supplementary file 1 — Supplementary Information [file 41698_2021_195_MOESM1_ESM.pdf]

Manufacturer / model name (n)	Total 308	Training set 258	Test set 50
**GE MEDICAL SYSTEMS**			
BrightSpeed S	2	2	0
Brivo CT385 Series	1	1	0
Discovery CT750 HD	40	33	7
HISpeed NX/i	1	1	0
LightSpeed Pro 16	1	1	0
LightSpeed VCT	32	27	5
LightSpped16	2	1	1
Optima CT660	4	3	1
Revolution EVO	1	1	0
**HITACHI MEDICAL CORPARATION**			
ECLOS	2	2	0
Presto	1	1	0
**Philips**			
ICT 256	9	8	1
ICT SP	2	1	1
Ingenuity CT	13	11	2
Access CT	1	1	0
Brilliance 6	1	1	0
Brilliance 64	15	12	3
Ingenuity Core	1	1	0
Ingenuity Core 128	1	1	0
IQon - Spectral CT	2	2	0
**PNMS**			
MX 16	2	2	0
**SIEMENS**			
Emotion	1	0	1
Emotion 16	2	2	0
Emotion 6	4	3	1
Emotion Duo	1	1	0
Scope	1	1	0
Sensation 16	5	5	0
Sensation 64	20	17	3
SOMATOM Definition	28	25	3
SOMATOM Definition AS	1	0	1
SOMATOM Definition AS+	44	37	7
SOMATOM Definition Edge	4	4	0
SOMATOM Definition Flash	18	16	2
SOMATOM Drive	1	1	0
SOMATOM Force	8	6	2
SOMATOM Scope	1	0	1
Volume Zoom	6	4	2
**TOSHIBA**			
Alexion	1	1	0
Aquilion	12	8	4
Aquilion ONE	4	3	1
Aquilion PRIME	9	8	1
Astelon	3	3	0

Supplementary Table 1. Manufacturers and model names of CT scanners for cases in an internal dataset.

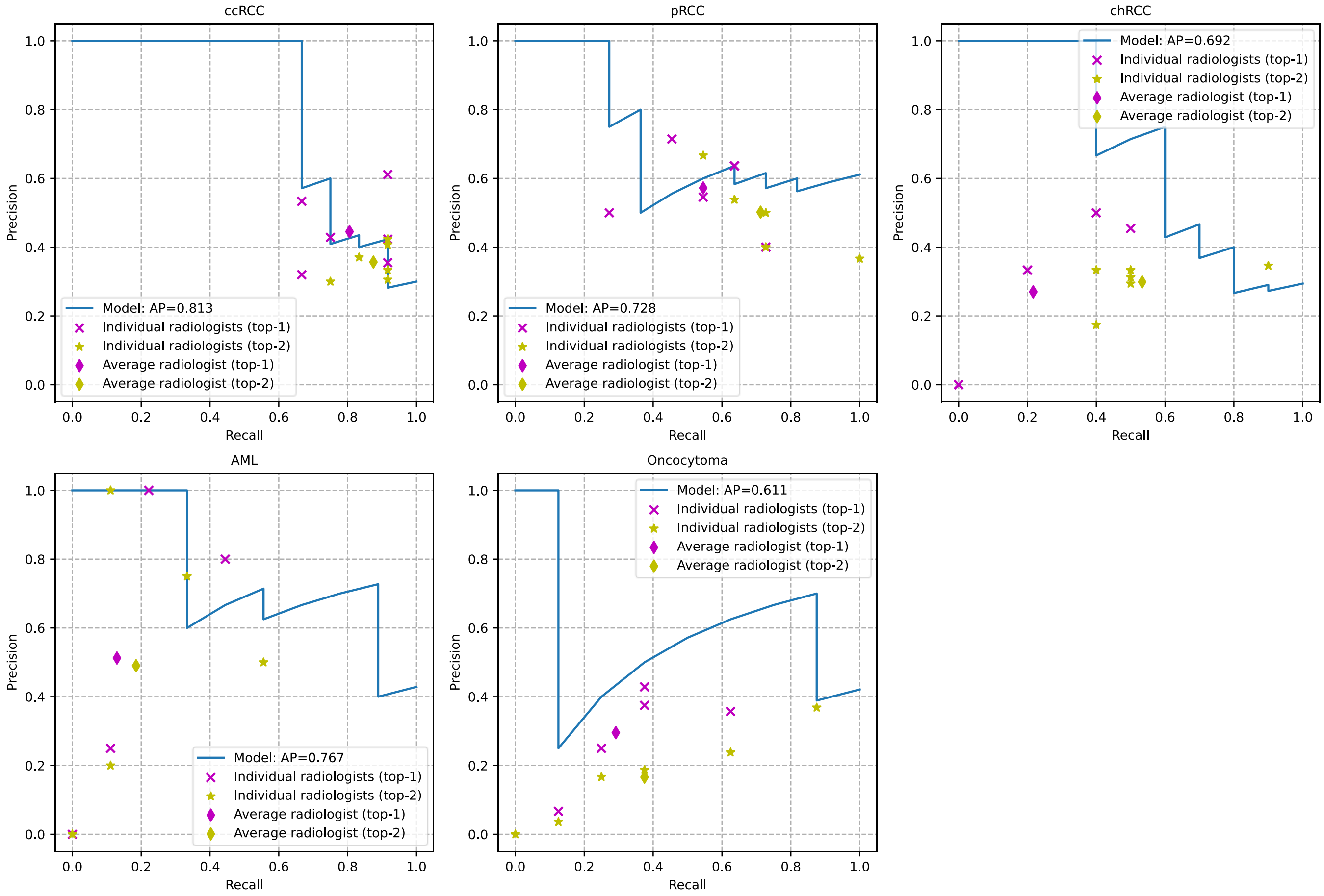

Supplementary Figure 1. Precision-recall curves for the model and radiologists on the internal validation dataset. The average precision (AP), i.e., the area under the precision-recall curve, is calculated for each curve.

	TCIA	Test set (full)	Test set (radiologist reviewed)
**Patients (n)**	263	184	40
**Age (years)**			
–40	21 (8.0)	13 (7.1)	5 (12.5)
40–50	47 (17.9)	34 (18.5)	10 (25.0)
50–60	77 (29.3)	56 (30.4)	13 (32.5)
60–70	63 (24.0)	44 (23.9)	8 (20.0)
70–	50 (19.0)	33 (17.9)	4 (10.0)
Unknown	5 (1.9)	4 (2.2)	-
**Gender**			
Female	88 (33.5)	58 (31.5)	6 (15.0)
Male	175 (66.5)	126 (68.5)	34 (85.0)
**Pathological subtype**			
ccRCC	229 (87.1)	163 (88.6)	19 (47.5)
pRCC	22 (8.4)	14 (7.6)	14 (35.0)
chRCC	12 (4.6)	7 (3.8)	7 (17.5)
**CT phases**			
Four-phase	45 (17.1)	45 (24.5)	5 (12.5)
Three-phase	139 (52.9)	139 (75.5)	35 (87.5)
Two-phase	27 (10.3)	-	-
Single-phase	52 (19.8)	-	-

Supplementary Table 2. Patient demographics and clinical characteristics of an independent dataset (TCIA). Data in parentheses show percentage.

Manufacturer / model name (n)	TCIA<br>263	Test set (full)<br>184	Test set (radiologist reviewed)<br>40
**GE MEDICAL SYSTEMS**			
Discovery STE	1	1	0
HiSpeed CT/i	6	0	0
LightSpeed Plus	11	9	1
LightSpeed Pro 32	1	0	0
LightSpeed QX/i	40	29	3
LightSpeed Ultra	23	15	2
LightSpeed VCT	25	17	5
LightSpeed 16	80	64	9
Optima CT660	1	1	1
**Philips**			
Brilliance 64	4	3	1
Gemini TF 64	1	1	1
Mx8000 IDT 16	2	2	1
Precedence 16P	1	1	0
Philips CT Aura	1	1	0
**SIEMENS**			
Biograph128	1	0	0
Emotion 16	1	0	0
Emotion 6	2	2	0
Sensation 16	11	6	3
Sensation 4	2	0	0
Sensation 40	1	0	0
Sensation 64	25	16	8
SOMATOM Definition	7	5	3
SOMATOM Definition AS+	6	5	1
SOMATOM PLUS 4	2	1	0
Volume Zoom	6	4	0
**TOSHIBA**			
Aquilion	2	1	1

Supplementary Table 3. Manufacturers and model names of CT scanners for cases in an independent dataset.

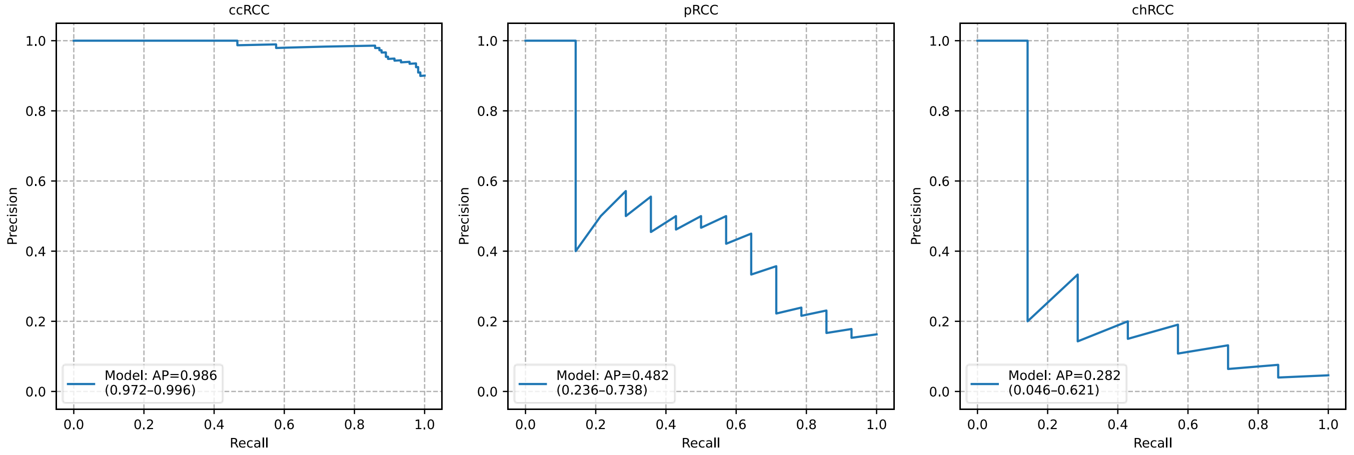

Supplementary Figure 2. Precision-recall curves of the model on the full TCIA test set. The average precision (AP), i.e., the area under the precision-recall curve, is calculated for each curve.

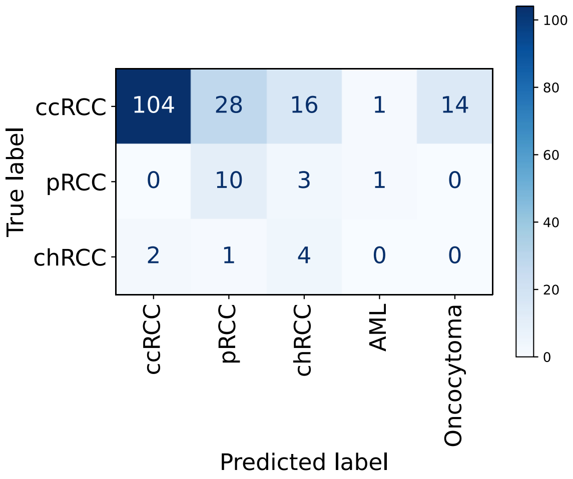

Supplementary Figure 3. Confusion matrix of the model on the full TCIA test set.

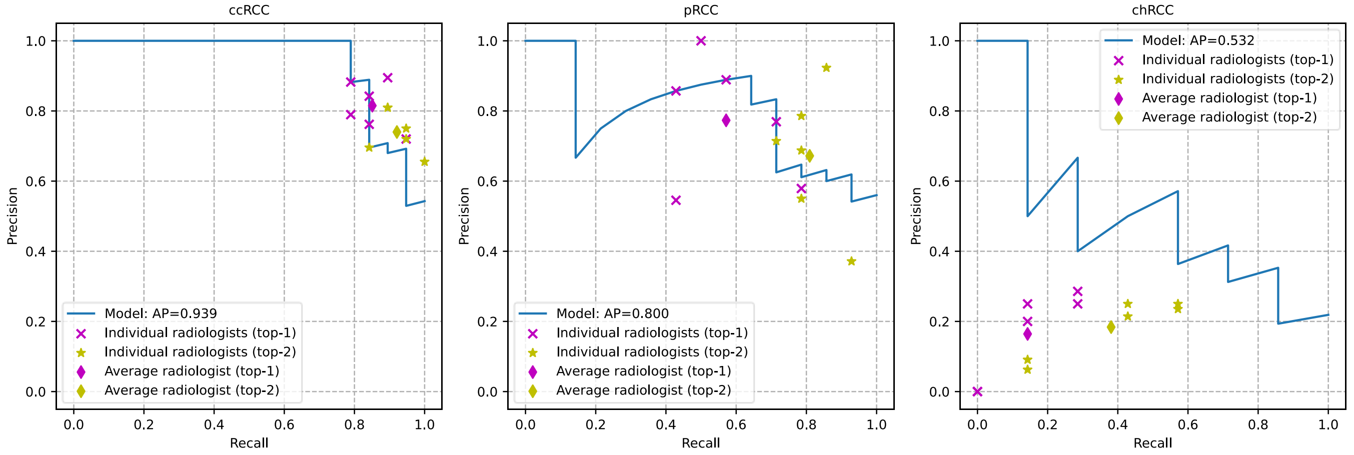

Supplementary Figure 4. Precision-recall curves for the model and radiologists on the reviewed TCIA dataset. The average precision (AP), i.e., the area under the precision-recall curve, is calculated for each curve.

a **Segmentation performance of the model (DSC)**

	Non-contrast	Arterial	Portal	Delayed
Kidney	0.965 ± 0.015	0.971 ± 0.011	0.971 ± 0.013	0.970 ± 0.014
Tumor	0.780 ± 0.197	0.875 ± 0.076	0.887 ± 0.073	0.887 ± 0.089

b **Multi-phase registration performance of the model (DSC)**

	Non-contrast	Arterial	Delayed
**Centering only**			
Kidney	0.883 ± 0.060	0.931 ± 0.039	0.917 ± 0.043
Tumor	0.736 ± 0.180	0.807 ± 0.133	0.772 ± 0.151
**Registration**			
Kidney	0.919 ± 0.028	0.945 ± 0.024	0.940 ± 0.022
Tumor	0.833 ± 0.105	0.853 ± 0.097	0.878 ± 0.059

Supplementary Table 4. Performance of segmentation and registration of the model. $\pm$ indicates mean $\pm$ standard deviation.
