# Supplementary material for: Deep learning for end-to-end kidney cancer diagnosis on multi-phase abdominal computed tomography
Source: NPJ Precis Oncol. 2021 Jun 18;5:54. doi: 10.1038/s41698-021-00195-y (PMC8213852; doi:10.1038/s41698-021-00195-y)
Supplement: Supplementary file 2 — Reporting Summary [file 41698_2021_195_MOESM2_ESM.pdf]

nature research

Corresponding author(s): Sung Hoo Hong; Sung-Jea Ko

Last updated by author(s): Jan 7, 2021

Reporting Summary

Nature Research wishes to improve the reproducibility of the work that we publish. This form provides structure for consistency and transparency in reporting. For further information on Nature Research policies, see our Editorial Policies and the Editorial Policy Checklist.

Statistics

For all statistical analyses, confirm that the following items are present in the figure legend, table legend, main text, or Methods section.

n/a	Confirmed	
☐	☒	The exact sample size (*n*) for each experimental group/condition, given as a discrete number and unit of measurement
☐	☒	A statement on whether measurements were taken from distinct samples or whether the same sample was measured repeatedly
☐	☒	The statistical test(s) used AND whether they are one- or two-sided<br>*Only common tests should be described solely by name; describe more complex techniques in the Methods section.*
☒	☐	A description of all covariates tested
☒	☐	A description of any assumptions or corrections, such as tests of normality and adjustment for multiple comparisons
☐	☒	A full description of the statistical parameters including central tendency (e.g. means) or other basic estimates (e.g. regression coefficient) AND variation (e.g. standard deviation) or associated estimates of uncertainty (e.g. confidence intervals)
☐	☒	For null hypothesis testing, the test statistic (e.g. *F*, *t*, *r*) with confidence intervals, effect sizes, degrees of freedom and *P* value noted<br>*Give P values as exact values whenever suitable.*
☒	☐	For Bayesian analysis, information on the choice of priors and Markov chain Monte Carlo settings
☒	☐	For hierarchical and complex designs, identification of the appropriate level for tests and full reporting of outcomes
☒	☐	Estimates of effect sizes (e.g. Cohen's *d*, Pearson's *r*), indicating how they were calculated

Our web collection on statistics for biologists contains articles on many of the points above.

Software and code

Policy information about availability of computer code

Data collection	nU PACS Viewer (version 1.0.0.29): FDA-approved fully featured PACS viewer. Used to collect reader study results.<br>ITK-SNAP (version 3.6.0): open source software used to label data
Data analysis	PyTorch (version 1.2): open source machine learning framework used to train deep learning models<br>Matplotlib (version 3.2.1): open source library for making plots<br>sklearn (version 0.22.2): open source library used for metrics such as AUC<br>nnUNet (https://github.com/MIC-DKFZ/nnUNet): open source framework used for development of segmentation models<br>Spatial transformer network (https://github.com/pytorch/tutorials/blob/master/intermediate_source/spatial_transformer_tutorial.py)<br>ResNet (https://github.com/pytorch/vision/blob/master/torchvision/models/resnet.py): open code used for classification models

For manuscripts utilizing custom algorithms or software that are central to the research but not yet described in published literature, software must be made available to editors and reviewers. We strongly encourage code deposition in a community repository (e.g. GitHub). See the Nature Research guidelines for submitting code & software for further information.

Data

Policy information about availability of data

All manuscripts must include a data availability statement. This statement should provide the following information, where applicable:

- Accession codes, unique identifiers, or web links for publicly available datasets
- A list of figures that have associated raw data
- A description of any restrictions on data availability

The TCIA dataset used for the external validation is publicly available at the TCIA data portal (https://www.cancerimagingarchive.net). The dataset from Seoul St. Mary's Hospital was used under license for the current study. Restrictions apply to the availability of this dataset and so it is not publicly available. However, data are available from the authors on reasonable request and with permission of Seoul St. Mary's Hospital.

Field-specific reporting

Please select the one below that is the best fit for your research. If you are not sure, read the appropriate sections before making your selection.

☒ Life sciences ☐ Behavioural & social sciences ☐ Ecological, evolutionary & environmental sciences

For a reference copy of the document with all sections, see nature.com/documents/nr-reporting-summary-flat.pdf

Life sciences study design

All studies must disclose on these points even when the disclosure is negative.

Sample size	We had to balance having enough data to train the algorithm while having enough data to validate the algorithm. We used a 84% training (258 cases) and 16% testing (50 cases) split which is a standard way of splitting datasets for deep learning research.
Data exclusions	For TCIA, we excluded cases with less than three CT phases.
Replication	We validated the performance of the proposed model on a completely independent dataset from a large public cancer imaging archive (TCIA).
Randomization	We randomly split the dataset into training set and test set. For the reader study on TCIA, we randomly selected 19 ccRCC cases to balance the number of test cases between subtypes. All the cases in the dataset were independent and non-repeating samples.
Blinding	Since our experiments are based on de-identified CT images, blinding is not necessary.

Reporting for specific materials, systems and methods

We require information from authors about some types of materials, experimental systems and methods used in many studies. Here, indicate whether each material, system or method listed is relevant to your study. If you are not sure if a list item applies to your research, read the appropriate section before selecting a response.

Materials & experimental systems

n/a	Involved in the study	
☒	☐	Antibodies
☒	☐	Eukaryotic cell lines
☒	☐	Palaeontology and archaeology
☒	☐	Animals and other organisms
☐	☒	Human research participants
☒	☐	Clinical data
☒	☐	Dual use research of concern

Methods

n/a	Involved in the study	
☒	☐	ChIP-seq
☒	☐	Flow cytometry
☒	☐	MRI-based neuroimaging

Human research participants

Policy information about studies involving human research participants

Population characteristics	we included 1,035 CT scans from 308 patients who underwent nephrectomy for renal tumors between 2003 and 2020. The dataset contains five major subtypes of renal tumors: oncocytoma (45 cases), AML (60 cases), chRCC (68 cases), pRCC (69 cases), and ccRCC (66 cases). All tumors have been pathologically confirmed by surgery. The patients were aged 20-40 years (29 cases), 40-50 years (73 cases), 50-60 years (96 cases), 60-70 years (71 cases), and 70-90 years (39 cases). The dataset included 141 males and 167 females. The tumor diameter ranged 1-2 cm (79 cases), 2-3 cm (84 cases), 3-4 cm (56 cases), 4-5 cm (33 cases), 5-6 cm (29 cases), 6-17 cm (27 cases).<br><br>The independent dataset from TCIA included 184 patients with at least three available CT phases. The dataset contains three

	subtypes of renal tumors: chRCC (7 cases), pRCC (14 cases), and ccRCC (163 cases). The patients were aged 20-40 years (13 cases), 40-50 years (34 cases), 50-60 years (56 cases), 60-70 years (44 cases), 70-90 years (33 cases), and unknown (4 cases). The dataset included 126 males and 58 females.
Recruitment	All participants signed an informed consent developed and were approved by the Seoul St. Mary's Hospital Institutional Review Board. The independent dataset was collected from the public repository (TCIA).
Ethics oversight	Seoul St. Mary's Hospital

Note that full information on the approval of the study protocol must also be provided in the manuscript.
